# 12 The Influence of Female Sex Hormones on Outcomes After Burn Injury

**DOI:** 10.1093/jbcr/irac012.016

**Published:** 2022-03-23

**Authors:** Kassandra K Corona, Juquan Song, Giovanna De La Tejera, Lyndon G Huang, Kendall Wermine, Sunny Gotewal, Tsola A Efejuku, Alejandro A Joglar, Phillip H Keys, Elvia L Villarreal, Shivan N Chokshi, Jasmine M Chaij, Alen Palackic, Amina El Ayadi, George Golovko, Steven E Wolf

**Affiliations:** University of Texas Medical Branch, Galveston, Texas; University of Texas Medical Branch, Galveston, Texas; University of Texas Medical Branch, Galveston, Texas; University of Texas Medical Branch, Galveston, Texas; University of Texas Medical Branch, Galveston, Texas; University of Texas Medical Branch, Galveston, Texas; University of Texas Medical Branch, Galveston, Texas; University of Texas Medical Branch, Galveston, Texas; University of Texas Medical Branch, Galveston, Texas; University of Texas Medical Branch, Galveston, Texas; University of Texas Medical Branch at Galveston, Galveston, Texas; University of Texas Medical Branch, Galveston, Texas; University of Texas Medical Branch, Galveston, Texas; University of Texas Medical Branch at Galveston, Galveston, Texas; University of Texas Medical Branch at Galveston, Galveston, Texas; University of Texas Medical Branch at Galveston, Galveston, Texas

## Abstract

**Introduction:**

The pathophysiological response to major trauma has gender dimorphism in outcomes associated with sex hormones levels. However, little is known of the effects of female hormones on outcomes in burn patients. Previous studies demonstrated exogenous estrogen alleviates hyper-inflammation after burn. We thus posit that female patients have fewer comorbidities depending on female hormone levels. We had two objectives: to investigate the role of female hormones on outcomes after burn and to investigate potential protective properties of exogenous hormone treatment on burned post-menopausal women.

**Methods:**

This study obtained data from the TriNetX research network with electronic medical records of de-identified patients. Both male and female patients who suffered burns were included from 2002-2020. The population was stratified to only include women over 45 on estrogen or progestin hormones taken within 6 months prior to injury and 1 month after injury to assure a menopausal state which occurs at age 45-55. Outcomes for mortality, sepsis, acute myocardial infarction, and acute kidney injury were measured within one day of injury to one month following injury. Odds ratios, risk difference, and risk ratios were calculated for outcome analysis after propensity-matched for race and ethnicity. A z-test for risk difference was performed. Statistical significance was defined at p < 0.05.

**Results:**

Compared to males, females grossly had a 28% risk reduction of 30-day mortality and a relative risk reduction for sepsis (26%), acute kidney failure (30), and myocardial infarction (29%) (p< .05). Additionally, female burns younger than the age 45 had risk reductions for mortality 5.4-fold within 3 months, 2.9-fold lower for sepsis, 17.5-fold lower for myocardial infarction, and 7.7-fold lower for acute kidney injury (p< .001). TriNetX identified 169,566 female burn patients, of which 2,683 were on estrogen and progestin and above 45 years old. Women over 45 on exogenous hormones had a 37% significant risk reduction in acute kidney failure when compared to the women over 45 not prescribed estrogen or progestin (p< .05).

**Conclusions:**

Female burn patients had better outcomes, while women over 45 had worse outcomes indicating the role of female sex hormone correlated to burn patient progress. The administration of estrogen and progestins for females above age 45 resulted in reduced risks for acute kidney failure after burn injury.

